# Recent advances in understanding hereditary spastic paraplegias and emerging therapies

**DOI:** 10.12703/r/10-27

**Published:** 2021-03-10

**Authors:** Pauline Lallemant-Dudek, Frederic Darios, Alexandra Durr

**Affiliations:** 1 Paris Brain Institute (ICM), Inserm U 1127, CNRS UMR 7225, Sorbonne Université, Paris, France; 2 Assistance Publique-Hôpitaux de Paris (AP-HP), Genetic Department, Pitié-Salpêtrière University Hospital, Paris, France

**Keywords:** Spastic paraplegia, cerebellar ataxia, spasticity

## Abstract

Hereditary spastic paraplegias (HSPs) are a group of rare, inherited, neurological diseases characterized by broad clinical and genetic heterogeneity. Lower-limb spasticity with first motoneuron involvement is the core symptom of all HSPs. As spasticity is a syndrome and not a disease, it develops on top of other neurological signs (ataxia, dystonia, and parkinsonism). Indeed, the definition of genes responsible for HSPs goes beyond the 79 identified *SPG* genes. In order to avoid making a catalog of the different genes involved in HSP in any way, we have chosen to focus on the HSP with cerebellar ataxias since this is a frequent association described for several genes. This overlap leads to an intermediary group of spastic ataxias which is actively genetically and clinically studied. The most striking example is *SPG7*, which is responsible for HSP or cerebellar ataxia or both. There are no specific therapies against HSPs, and there is a dearth of randomized trials in patients with HSP, especially on spasticity when it likely results from other mechanisms. Thus far, no gene-specific therapy has been developed for HSP, but emerging therapies in animal models and neurons derived from induced pluripotent stem cells are potential treatments for patients.

## Introduction

Hereditary spastic paraplegias (HSPs) are a group of rare, inherited, neurological diseases characterized by broad heterogeneity in terms of both their clinical manifestations and genetic causes. HSPs are characterized by degeneration of the corticospinal tract, predominantly of the first motoneuron alone but often involving the second motoneuron^1^. The main symptom is progressive bilateral lower-limb spasticity as a component of pyramidal syndrome.

Pyramidal syndrome is largely a component of more frequently occurring neurological diseases, such as progressive forms of multiple sclerosis or slowly evolving forms of amyotrophic lateral sclerosis, often resulting in delayed genetic diagnosis of HSP^2^. Without genetic diagnoses, it is difficult to establish the prevalence of HSP. The estimated prevalence based on genetic or clinical diagnoses (or both) from a recent Swedish study is 2.4 out of 100,000^3^. This prevalence is near those found in a previous international literature review (2.2 out of 100,000)^4^ and a Portuguese population-based study (4.1 out of 100,000)^5^. One meta-analysis included 13,570 patients with a genetic diagnosis of the most frequently occurring genes (*SPAST*, *REEP1*, *ATL1*, *SPG11*, *SPG15*, *SPG7*, *SPG35*, *SPG54*, and *SPG5*)^6^. Unsurprisingly, SPG4/*SPAST*-HSP represents the most frequent overall (25%)^6^ as an autosomic dominant transmission, followed by *REEP1* and *ATL1*. The most common autosomic recessive form is *SPG11*. The relative prevalences vary greatly among populations. The frequency of each gene is influenced by the degree of consanguinity (autosomic recessive bias according to the population screened) and the existence of a founder effect. Furthermore, multilocus and multigenic inheritance as in inherited axonopathies could be the underlying genetic cause, resulting in complex transmission patterns that are more difficult to be recognized^7^.

SPG4/*SPAST*-HSP is characterized by extreme inter- and intra-familial variability for the age at onset, ranging from birth to the eighth decade, based on recently updated data following analysis of the world’s largest SPG4/*SPAST*-HSP patient cohort (n = 842)^8^. Analysis of this large cohort made it possible to firmly establish a bimodal distribution for the age at onset for SPG4/*SPAST*-HSP, and a first major peak occurs before 10 years of age and a second smaller peak occurs between the third and fifth decades. Furthermore, such bimodality reflected the nature of the *SPAST* causative variants. Indeed, missense variant carriers were characterized by disease onset that was significantly earlier than those of truncating variant carriers. This highlighted, for the first time, a clear genotype–phenotype correlation^8^. Intrafamilial variation of the age at onset due to the same shared causative *SPAST* variant has yet to be unraveled and is most likely due to genetic or environmental modifiers (or both). To date, *SPAST* Exon 1 variant c.131C>T/p.(Ser44Leu) appears to be the most well-documented SPG4/*SPAST*-HSP genetic modifier, leading to marked lowering of the age at onset when carried in combination with a major pathogenic *SPAST* mutation^9,10^.

The age-at-onset phenotype appears to be narrower for other HSP types. SPG3A/*ATL1*-HSP, the second most frequent dominant HSP, nearly always begins before the age of five years. Both SPG4/*SPAST*-HSP and SPG3A/*ATL1*-HSP are purely pyramidal and are associated with considerable sensory loss at the ankles in the former. The distribution of the age at onset for relatively frequent autosomal recessive forms of HSP is narrower, such as that described for SPG11, which represents 8 to 18% of HSP cases^6^ and has an early onset (before 10 years of age).

What genes are considered to be responsible for HSP? Certainly, more than simply the *SPG* genes. In a recent review, 79 *SPG* genes were identified^11^. Cerebellar ataxia is often associated, leading to an individualized group of spastic ataxias. There is a definite overlap between the two disease groups, both clinically and genetically^12^.

## Spastic ataxias

The most striking example for overlap between HSP and ataxias is *SPG7*. The first description was based on two Italian pure-HSP families and one French family, which included three sisters with a pure spastic phenotype on examination, but showing a more complex disease upon imaging and fundus examination, with cerebellar atrophy and optic atrophy^13^. Paraplegin, encoded by *SPG7*, is a member of the AAA (ATPases associated with a variety of cellular activities) protein family, which is localized to the inner mitochondrial membrane^14,15^. Paraplegin assembles with AFG3L2 (SCA28) to form the oligomeric mitochondrial AAA protease complex, which is involved in protein maturation and degradation^16–21^. Interestingly, *SPG7* has been shown to be causative for up to 19% of undiagnosed cerebellar ataxias^22,23^. We were able to analyze the largest *SPG7* cohort (241 patients), assembled through the European SPATAX network (https://spatax.wordpress.com/). We explored genotype–phenotype correlations in this cohort. We found that patients with loss-of-function variants have a more complicated phenotype, with spasticity. On the contrary, patients carrying at least one Ala510Val variant showed more frequent cerebellar ataxia and later onset^24^. Although cerebellar atrophy is known to be a hallmark of *SPG7*, a specific pattern of gray matter cerebellar atrophy that affects long-distance regions of the brain was recently shown to be associated with cognitive and social-ability impairment^25^. *SPG7* is not the only SPG associated with cerebral atrophy.

Aside from *SPG7*, there are other examples of overlap between cerebellar ataxia and spastic paraplegias (Table 1). Among polyglutamine expansion spinocerebellar ataxias, which share a mutational mechanism with other polyglutamine expansion diseases, such as Huntington disease and spinal bulbar muscular atrophy, the presence of spasticity is very common^26^. The presence of a pyramidal syndrome is linked to the size of the CAG repeat expansion. The longer the CAG repetition is, the more important the spasticity is in addition to the cerebellar ataxia. Neuropathological features showed the involvement of the upper and lower motor neurons (that is, the corticospinal tracts or the anterior horn degeneration in SCA1, 2, 3, and 7 or both)^53^.

**Table 1.** Most frequent examples of overlap between spastic paraparesis and cerebellar ataxia according to their transmission and clinical presentation.

Locus / *Gene*	Transmission	Clinical features	Physiopathology / Treatment
*Spastic paraparesis or cerebellar ataxia according to the transmission or other factors (unknown)*			
SPG7 / *SPG7*^22–24,27–30^	**AR, AD?**	**AR** spastic paraparesis with cerebellar atrophy, optic atrophy, ptosis, ophthalmoplegia Or Cerebellar ataxia with pyramidal signs, ophthalmoplegia, parkinsonism **AD** isolated cerebellar atrophy (not confirmed)	*SPG7* encodes paraplegin, an adenosine triphosphate (ATP)‐ dependent zinc metalloproteinase that assembles with the mitochondrial metallo-proteinase AFG3L2 (SCA28).
SPG46 / *GBA2*^31–33^	**AR**	Spastic paraparesis, other pyramidal signs, muscle weakness, pseudobulbar dysarthria, cognitive impairment, mental retardation, congenital cataracts, nystagmus, hearing loss, head tremor, peripheral neuropathy, pes cavus, scoliosis, infertility, small testicles, hypogonadism in males Or Cerebellar ataxia with kinetic and static ataxia, pyramidal syndrome and external ophtalmoparesis, with jerky pursuit and slow saccades	*GBA2* encodes B-glucosidase 2, which cleaves the sphingolipid glucosylceramide into glucose. and ceramide, leading to further metabolism to sphingomyelin.
SPG39 / *PNPLA6*^27,34,35^	**AR**	Spastic paraparesis, peripheral neuropathy, visual impairment due to chorioretinal dystrophy, nystagmus, distal muscle atrophy, intention tremor, cognitive impairment, hypogonadism, trichomegaly, short stature, hypothyroidism Or Cerebellar ataxia and/or hypogonadism	*PNPLA6* encodes the neuropathy target esterase, which belongs to a protein family of nine patatin-like phospholipase domain-containing proteins. The most important functional domain is the EST domain. This domain is needed to transform phosphatidylcholine in glycerophosphocholine which is a precursor of acetylcholine (nervous system neurotransmitter).
*Cerebellar ataxia at the forefront*			
*CYP27A1*^36–38^ Cerebrotendinous xanthomatosis (CTX)	**AR**	Cerebellar ataxia, spastic paraplegia, tuberous skin and tendon xanthomas, xanthelasmas, cataracts, chronic diarrhea, cognitive decline, psychiatric symptoms, peripheral neuropathy, parkinsonism, pseudobulbar palsy, seizures	*CYP27A1* encodes the mitochondrial enzyme sterol 27-hydroxylase, involved in bile-acid synthesis. *CYP27A1* mutations lead to decreased synthesis of bile acid. Early diagnosis and long-term treatment with chenodeoxycholic acid improve neurological symptoms and contribute to a better prognosis.
*FXN*^39–41^ Friedreich’s Ataxia (FRDA)	**AR**	Cerebellar and sensitive ataxia, square-wave jerks, optic atrophy, hearing loss, peripheral sensory neuropathy, spasticity, scoliosis, hypertrophic cardiomyopathy, diabetes Spinal cord atrophy, absence of cerebellar atrophy Homozygous for GAA trinucleotide repeat expansions: common Friedreich’s ataxia. Compound heterozygotes for a GAA expansion with missense mutations (D122Y and G130V): mild cerebellar ataxia, with early-onset spastic gait, slow progression	*FXN* encodes a mitochondrial protein that belongs to the FRATAXIN family. The protein regulates mitochondrial iron transport and respiration. The expansion of the intronic trinucleotide repeat GAA from 8 to 33 repeats to more than 90 repeats results in Friedreich’s ataxia. Several treatments have been tested. For example, deferiprone has an acceptable safety profile for the treatment of patients with FDRA and low doses may reduce disease progression. Trials are in progress. MOXIe is a randomized, placebo-controlled, double-blind parallel-group study to evaluate the safety and efficacy of various doses of omaveloxolone. It may activate Nrf2 and induce the expression of antioxidant target genes.
*NPC1*^42^ Niemann–Pick disease type C	**AR**	Cerebellar ataxia, developmental regression, psychiatric symptoms, loss of speech, vertical supranuclear gaze palsy, dystonia, intention tremor, spasticity, seizures, hepatosplenomegaly, cholestatic jaundice, gelastic cataplexy	*NPC1* encodes a large protein that resides in the limiting membrane of endosomes and lysosomes and mediates intracellular cholesterol trafficking via binding of cholesterol to its N-terminal domain. Low density lipoproteins are carried via this protein and relaunched as free cholesterol. Miglustat treatment has good responses on patients with less severe condition at the beginning of the treatment.
*POLR3A*^43^	**AR**	Cerebellar ataxia, spasticity, developmental regression, optic atrophy, nystagmus, abnormal saccades, vertical gaze palsy, postural tremor, dystonia, seizures, peripheral neuropathy, oligodontia, hypogonadotropic hypogonadism, short stature	*POL3RA* encodes a catalytic component of RNA polymerase III, which synthesizes small RNAs. The encoded protein also acts as a sensor to detect foreign DNA and trigger the innate immune response.
*POLR3B*^43^	**AR**	Cerebellar ataxia, spasticity, cognitive decline, nystagmus, abnormal saccades, vertical gaze palsy, myopia, tremor, oligodontia, short stature, hypogonadism	*POLR3B* encodes the second largest subunit of RNA polymerase III, the polymerase responsible for synthesizing transfer and small ribosomal RNAs in eukaryotes. The largest subunit and the encoded protein form the catalytic center of RNA polymerase III.
SCAR18/*GRID2*^44^	**AR, AD,** ***de novo***	**AR** Congenital ataxia with pyramidal signs, developmental delay, cognitive impairment, strabismus, nystagmus, oculomotor apraxia, tonic upgaze, retinopathy, muscle atrophy, joint contractures, scoliosis **AD** Very mild ataxia, one family with heterozygous c.1966C. G/p.Leu656Val variant. Hearing loss and cognitive impairment is prominent. In the homozygous state: congenital ataxia. Two heterozygous de novo variants c.1960G.A/p.Ala654Thr, c.1961C.A/p.Ala654Asp: congenital ataxia	*GRID2* encodes the glutamate receptor d2 (GluRD2) protein, which forms a complex with cerebellin-1 to create a synapse organizer in the cerebellum.
SCAR16, SCA48/ *STUB1*^45,46^	**AR, AD**	**AR** Spastic ataxia, nystagmus, external ophthalmoplegia, myoclonus, cognitive impairment, peripheral neuropathy, reduced vibration sense in lower limbs, hypogonadism **AD** Spastic ataxia with predominant cognitive impairment (CCAS)	STIP1 homology and U-Box containing protein 1 (*STUB1*) encodes the CHIP protein, containing tetratricopeptide repeats and a U-box that functions as a ubiquitin ligase/co-chaperone, involved in the cellular protein quality-control system.
SCAR1, AOA2/ *SETX*^43^	**AR**	Spastic ataxia spastic, oculomotor apraxia, dystonia, chorea, axonal peripheral neuropathy, scoliosis	Senataxin (SETX) acts as an RNA/DNA helicase that resolves R-loops.
*Spasticity at the forefront*			
*FOLR1*^47,48^	**AR**	Spastic ataxia, developmental regression, visual disturbances, sensorineural hearing loss, chorea, generalized tonic-clonic, atonic, and myoclonic seizures	*FOLR1* encodes a protein existing either fixed on membranes via a glycosyl-phosphatidylinositol linkage or in a soluble form. This folate-receptor binds folic acid and transports its derivated into cells. Folinic acid therapy.
SPG76/*CAPN1*^49^	**AR**	Cerebellar ataxia with severe spasticity and dysarthria, overall slowness	*CAPN1* encodes calpain-1, of which the absence results in inhibition of the Akt pro-survival pathway in developing granule cells. Treatment with a potential Akt activator.
SPAX5 SCA28/ *AFG3L2*^43–46,49,50^	**AR, AD**	**AR** Spastic ataxia, oculomotor apraxia, dystonia, myoclonus, myoclonic epilepsy, generalized tonic-clonic seizures, distal muscle atrophy, peripheral neuropathy **AD** Very mild ataxia, with external ophthalmoplegia and ptosis	Variants lead to decreased AFG3L2 activity, with accumulation of mitochondria-encoded proteins, causing unable mitochondrial fusion and calcium buffering less efficient (by modification of OMA1 and OPA1 process).
SPAX6, ARSACS/ *SACS*^51,52^	**AR**	Spastic ataxia, delayed walking development, demyelinating peripheral neuropathy, nystagmus, impaired smooth pursuit, dystonia, erectile dysfunction, urinary incontinence, retinal striation	*SACS* encodes the sacsin protein, which includes a UbL domain at the N-terminus, a DnaJ domain, and a HEPN domain at the C-terminus. Sacsin may protect against mutant ataxin-1 and may regulate the effects of other ataxia proteins.

AD, autosomic dominant; AR, autosomic recessive.

Like *SPG7*, mutations in *GBA2* and *PNPLA6* can result in either spastic paraplegia or cerebellar ataxia with an autosomal recessive transmission mode^31–34^. Other genes have been identified to promote diseases characterized by cerebellar ataxia with spasticity. Eight are known, such as Cerebrotendinous xanthomatosis, Friedreich’s ataxia, and Niemann–Pick disease type C. In less frequent diseases, such as those caused by mutations of *SCAR18*, the intensity of clinical presentation and ataxia depends on the transmission mode^44^. Spasticity is at the forefront of several other genetic diseases related to *FOLR1*, *CAPN1*, *AFG3L2*, and *SACS*^43,47–52^. Knowledge of these diseases and their identification are indispensable for providing specific treatment.

## Management of HSP

### Magnetic resonance imaging and HSP diagnosis

New neuroimaging findings allow precise HSP diagnosis on cerebral magnetic resonance imaging (MRI). For example, in SPG11, most patients have a thin corpus callosum^54^ and another sign is known as the “ear of the lynx”^55^. This sign corresponds to long T1 and T2 values in the forceps minor of the corpus callosum, which appears hyperintense on FLAIR (fluid-attenuated inversion recovery) and hypointense on T1-weighted images. Furthermore, new MRI techniques identified specific gray and white matter damage^56^. Neuropsychological exam and neuroimaging were performed on 25 SPG11 patients. This study allows us to describe a widespread pattern of damage in white matter probably linked to cognitive deficiency, described in this population, whereas progressive degeneration of multiple gray matter structures and spinal cord seems to be correlated with disease duration.

Another HSP well described by MRI is Pelizaeus–Merzbacher disease (L1CAM/*SPG1-HSP*). It is characterized by hypomyelination of brain stem and corticospinal tract on internal capsule^57^. The hypomyelination underlines another overlap existing between HSP and leukodystrophies. Hypomyelinating disorders are observed in different genetic diseases. But the MRI pattern gives a clue to identify pathologies as Pelizaeus–Merzbacher disease, infantile GM1 and GM2 gangliosidosis, or fucosidosis^58^.

### Symptomatic treatment

Symptomatic treatments for ataxia and HSP are complex and need long-term engagement from the patient and caregivers^59^. The first step is to implement rehabilitation therapies to preserve functions and develop compensations. Prevention of ataxia complications is necessary below the ataxia evolution. A multidisciplinary team for evaluation and management is essential to accompany patients over the long term at best and sometimes up to palliative care. For example, oropharyngeal dysphagia is a common symptom of the bulbar syndrome. It is one of the first symptoms in cerebellar ataxia which affect quality of life^60,61^ and can lower life expectancy. It can occur in complications such as malnutrition, dehydration, and aspiration-related pneumonia. Therapy carried out by speech and language therapists has a positive significant outcome^61^. Another cerebellar symptom is the downbeat nystagmus. Different GABAergic substances, such as the 3,4-diaminopyridine and the 4-aminopyridine, have been tested. They have no major side effects and are well tolerated for a moderate success^62^. Baclofen is another GABAergic substance used against upbeat nystagmus.

Baclofen is also used in HSP as an oral anti-spastic. There are no specific therapies for the treatment of HSP. Treatment for spasticity of various origins is well recognized but does not follow national or international recommendations, as only a very small number of studies have evaluated symptomatic treatment^63^, in HSP. Studies from 2016 have evaluated oral anti-spastic treatment in children with cerebral palsy and concluded that randomized trials are needed^64,65^. Nonetheless, oral treatment such as levodopa has shown their efficacy on specific subgroups of HSP (*SPG11* mimicking dopa-responsive dystonia and variant phenotype linked to *GCH1* mutations)^66,67^. In addition to being treated by baclofen or levodopa, spasticity is treated by intramuscular injection with botulinum toxin type A. This treatment is recommended for focal spasticity as well as dystonic postures in many neurological diseases (e.g., multiple sclerosis^68^ and cerebral palsy^69^). There has been only one retrospective study to evaluate the efficacy of combined treatment, consisting of botulinum toxin injection and intensive physical therapy for HSP^70^. Recruitment over five years included a small number of patients, including those with various genetic entities—SPG4 n = 5, SPG5 n = 1, SPG7 n = 1, SPG8 n = 2, SPG11 n = 1, SPG72 n = 1—and seven without a genetic diagnosis. All patients were given intramuscular injections of botulinum toxin followed by intensive physical therapy sessions (10 individualized sessions lasting two hours). After three months, they reported significantly reduced disease severity by the Spastic Paraplegia Rating Scale, reduced muscle tone (Modified Ashworth scale), and increased walking speed. However, they were unable to untangle the effect of injection from that of intensive physical therapy. Furthermore, there was no specific botulinum toxin injection protocol. The muscles injected, botulinum type, and administration of doses were left to the discretion of the physicians. This study also highlighted the difficulty of recruiting a large number of genetically and clinically homogenous patients. Several other symptomatic treatments have been tested and described through case reports or a cohort with very few patients. The treatments tested are, for instance, transcranial magnetic stimulation^71^, spinal cord stimulation^72^, and specific rehabilitation protocol^73^.

HSP spasticity is a type of stiffness that emerges mainly during movement, such as walking or “catch and release” maneuvers. This aspect must be considered in establishing HSP-specific treatment for spasticity versus that for spasticity of any origin. At rest, spasticity is considerably reduced or absent, especially during the initial stages of the disease. This specific dynamic pattern is underlined by two findings: (i) dying back and regeneration of the corticospinal tract (high density of small fibers with thin myelin fibers found in SPG4)^8^ and/or (ii) a lack of coordination of cerebellar origin between agonist and antagonist muscles during movement. Such a lack of coordination was revealed by gait analysis of 23 HSP patients versus that of 23 controls^74^. In that study, both knee and ankle muscles showed increased coactivity indices, and energetic parameters were higher for patients with HSP. The severity of the spasticity increased with coactivation, suggesting a lack of coordination: the abnormal activation of antagonist muscles obliged the agonist muscles to develop greater strength, resulting in spasticity.

There are only a few studies concerning how patients cope with their disease and the effectiveness of treatment from their point of view. One such study asked patients with HSP about their life with spasticity^75^. The patients felt ashamed of their disability and had difficulties tolerating their treatment. The symptoms of fatigue, depression, day-to-day fluctuations, and back pain^76^ are currently managed by patients but require more medical attention. A self-administered questionnaire completed six to eight times a day for a week by 35 patients with HSP showed that physical therapy and physical activity represented a very small proportion of their daily activities, and some reported no such activities despite their known usefulness against spasticity^77^.

Urinary and fecal disturbances, which are present and very frequent for 75% of patients with HSP, have been even less explored despite their recognized adverse impact on the quality of life of patients with HSP^78^. Women are more often affected than men^78^. Furthermore, there have been no studies on fecal disturbances and their effect on spasticity. These difficulties are well described in Parkinson disease and multiple sclerosis^79,80^, for example, but rarely in HSP^81^.

### Hope for a gene-specific therapy?

No gene-specific therapy for HSP has been developed thus far, but physiopathological studies performed in animal models or neurons derived from induced pluripotent stem cells have provided potential therapeutic targets for some forms of HSP.

The first treatment trials took place for SPG5, an autosomal recessive HSP, caused by pathogenic variants in *CYP7B1*, which encodes oxysterol-7alpha-hydroxylase. This enzyme is involved in cholesterol degradation and leads to the accumulation of neurotoxic oxysterol (27-OH hydroxycholesterol) when altered^82^. Two treatment trials have been carried out, both aiming to lower 27-hydroxycholesterol levels in patient serum as a read-out; they achieved the lowering of oxysterol levels but with no clinical benefit.

A replacement therapy was recently investigated by administrating CYP7B1 mRNA in a *CYP7B1* knockout mouse model^83^. Forty-eight hours after the injection of mouse or human CYP7B1 mRNA, 25-hydroxycholesterol levels were considerably lower in the liver and serum and modestly lower in the brain, whereas 27-hydroxycholesterol levels were lower only in the serum. It was not possible to test whether this treatment was associated with an improvement in the motor phenotype as the *CYP7B1* knockout mouse model does not show any obvious motor symptoms^83^. Impaired lipid metabolism has also been observed in a mouse model of SPG11, in which the accumulation of lipids in lysosomes has been shown to contribute to neurodegeneration. The absence of the *SPG11* product, spatacsin, impairs cholesterol trafficking and leads to the accumulation of certain glycosphingolipids and gangliosides in lysosomes^84,85^. Decreasing ganglioside levels using miglustat improved the motor phenotype in a *SPG11* zebrafish model, suggesting that this could be a viable therapeutic strategy. However, miglustat poorly crosses the blood–brain barrier and it would be informative to test whether an alternative strategy to decrease ganglioside levels in the brains of *Spg11* knockout mice can improve the motor or cognitive symptoms that have been observed^86^. As SPG11 patients generally present their first symptoms before 10 years of age, it has been proposed that aside from neurodegeneration, altered brain development may contribute to the disease^87^. Consistent with this hypothesis, models derived from induced pluripotent stem cells of SPG11 patients show reduced proliferation of neuronal progenitors, impaired neurogenesis, and impaired neuronal differentiation^88,89^. These phenotypes have been shown to result from impaired GSK3β/β-catenin signaling^90^. Tideglusib, a US Food and Drug Administration–approved GSK3β inhibitor, has been shown to restore the proliferation of neuronal progenitors and neuronal differentiation and correct the abnormal growth of SPG11 cortical organoids^89–91^. Tideglusib could thus be a therapy candidate for SPG11 patients, but its action on the developmental phase of the disease may preclude a beneficial effect in symptomatic patients. Preclinical and clinical studies are required to determine the efficacy of tideglusib as a therapy for SPG11 patients.

The product of *SPG4*, spastin, is a microtubule-severing protein. The downregulation of spastin or the expression of mutant spastin in *Drosophila* impairs locomotor performance and, at a subcellular level, leads to the stabilization of microtubules in synapses^92^. Treatment with the microtubule-targeting drug vinblastine reverts the synaptic phenotype in these *Drosophila SPG4* models^92^. Similarly, neurons derived from *Spg4* knockout mice show axonal swelling associated with impaired microtubule dynamics^93^. Microtubule-targeting drugs, such as nocodazole, Taxol, or vinblastine, restore the pathological phenotype observed in *Spg4*-knockout neurons. Similar results have been obtained in neurons derived from induced pluripotent stem cells of SPG4 patients, supporting the idea that microtubule-targeting drugs could be of therapeutic interest for SPG4. However, this therapeutic strategy has yet to be translated into preclinical or clinical studies.

Spastin, together with atlastin-1 (*SPG3*) and REEP1 (*SPG31*), has been shown to play a role in linking microtubules to the endoplasmic reticulum (ER), thus controlling its shape^91^. *Caenorhabditis elegans* and *Drosophila* models of *SPG4* also show signs of ER stress, and drugs known to modulate ER stress are able to improve motor symptoms in these animal models^95^. ER stress has also been observed in a *Drosophila* model of SPG31, and naringenin, a drug that protects cells from ER stress, is able to improve motor activity and life span in this model^96^. These studies highlight the role of ER stress as a potential mechanism that contributes to the physiopathology of HSP. ER stress could be used as a biomarker and therapeutic target for some forms of the disease. However, the presence of ER stress has thus far been observed only in invertebrate HSP models and has not yet been validated in mammalian models of SPG4 or SPG31 or in patients. This knowledge is critical before envisaging the targeting of ER stress as a therapeutic strategy.

These data show that physiopathological studies can lead to the identification of therapeutic strategies for various forms of HSP. However, the challenge will be to develop a specific treatment for each HSP subtype, given the large heterogeneity of these diseases. The alteration of ER stress in both SPG4 and SPG31 models suggests that common physiopathological mechanisms and thus common therapeutic targets could emerge, grouping several genetic subtypes. However, they will need to be validated in each subgroup of patients with HSP and will rely on the development of specific biomarkers. Rare diseases merit specific trials to develop new treatment strategies^97^, especially the use of homogeneous cohorts. There is a lack of natural history data, especially longitudinal biomarker analysis. To overcome these limitations, collaborative work with multinational cohorts is needed and identification of biomarkers should start.

## Conclusions

The genetic heterogeneity of HSP is continuing to be unraveled by the discovery of new genes. Clear correlations between genotype and age at onset have been established, and a search for genetic or environmental modifiers will be necessary. Certain genes are shared between HSP and ataxias, and *SPG7* is the leading example. For patients with a negative HSP panel result, genes responsible for other overlapping disorders need to be considered and after the testing for SPG4 exome sequencing is justified. In addition, pathological repeat expansion disorders could be ruled out.

The problem of heterogeneity also arises in studies on symptomatic treatment. Indeed, there is no consensus. Most studies have involved too few patients, sometimes with broad clinical and genetic heterogeneity, to allow global and generalizable conclusions. Thus, HSP is treated on the basis of the results of studies of other diseases without considering the characteristics of this population. This is particularly true in terms of spasticity. Indeed, HSP spasticity likely has a mechanism different from that of inflammatory diseases in particular. If the lack of coordination is at the origin of such spasticity, treatments must be rethought. This hypothesis strengthens the close links between HSP and spastic ataxia and opens up new avenues for the management of these diseases.
